# Effect of Natural Aging on the Stress Corrosion Cracking Behavior of A201-T7 Aluminum Alloy

**DOI:** 10.3390/ma13245631

**Published:** 2020-12-10

**Authors:** Mien-Chung Chen, Ming-Che Wen, Yang-Chun Chiu, Tse-An Pan, Yu-Chih Tzeng, Sheng-Long Lee

**Affiliations:** 1 Institute of Material Science and Engineering, National Central University, Taoyuan 320, Taiwan; mianzhongchen@gmail.com (M.-C.C.); albert77918@yahoo.com.tw (Y.-C.C.); peterpan.ck@gmail.com (T.-A.P.); 2 Department of Mechanical Engineering, National Central University, Taoyuan 320, Taiwan; j0e9rr3y0@gmail.com; 3 Department of Power Vehicle and Systems Engineering, Chung-Cheng Institute of Technology, National Defense University, Taoyuan 334, Taiwan; a0932467761@gmail.com

**Keywords:** Al-Cu-Mg-Ag alloy, natural aging, stress corrosion cracking, SSRT, PFZ

## Abstract

The effect of natural aging on the stress corrosion cracking (SCC) of A201-T7 alloy was investigated by the slow strain rate testing (SSRT), transmission electron microscopy (TEM), scanning electron microscopy (SEM), differential scanning calorimetry (DSC), conductivity, and polarization testing. The results indicated that natural aging could significantly improve the resistance of the alloys to SCC. The ductility loss rate of the unaged alloy was 28%, while the rates for the 24 h and 96 h aged alloys were both 5%. The conductivity of the as-quenched alloy was 30.54 (%IACS), and the conductivity of the 24 h and 96 h aged alloys were decreased to 28.85 and 28.65. After T7 tempering, the conductivity of the unaged, 24 h, and 96 h aged alloys were increased to 32.54 (%IACS), 32.52 and 32.45. Besides, the enthalpy change of the 24 h and 96 h aged alloys increased by 36% and 37% compared to the unaged alloy. The clustering of the solute atoms would evidently be enhanced with the increasing time of natural aging. Natural aging after quenching is essential to improve the alloy’s resistance to SCC. It might be due to the prevention of the formation of the precipitation free zone (PFZ) after T7 tempering.

## 1. Introduction

A201 (Al-4.5Cu-0.3Mg-0.7Ag) is a heat treatable aluminum alloy, which has the highest strength among the casting aluminum alloys, so it has been used in the aerospace and military industries for many years [1] The primary strengthening phases of A201 are θ’ and Ω, both having a similar composition to that of CuAl_2_ [2,3]. The crystal structure of θ’ is tetragonal and with a = b = 0.414 nm and c = 0.580 nm, forming large rectangular or octagonal plates parallel to the {100}_α_ plane of the matrix α phase [4]. The Ω phase has a face-centered orthorhombic structure, with a = 0.496 nm, b = 0.859 nm and c = 0.848 nm, which forms hexagonal plate-like precipitates on the {111}_α_ plane of the matrix α phase [5,6,7,8].

To enhance the mechanical properties, especially the tensile strength, a T6 temper treatment (solution heat treated then artificially aged) is usually applied in heat treatable alloys [1]. However, for high strength Al-Cu-Mg (2XX series) or Al-Zn-Mg-Cu (7XXX series) alloys, T6 temper treatment is not recommended because it will increase the susceptibility of the alloy to stress corrosion cracking (SCC) [9,10,11,12,13,14]. SSC can occur when alloys are simultaneously subjected to stress and corrosive environments. Burleigh [15] specified three SCC mechanisms for aluminum alloys, including anodic dissolution, hydrogen-induced cracking, and the brittle passive film’s rupturing. Speidel [16] have indicated that anodic dissolution is the primary mechanism of SCC in Al-Cu alloys. Misra [17] showed that Al-Cu alloys formed a precipitate free zone (PFZ) along the grain boundary following artificial aging, and this zone acts as an anode relative to the base of alloy. Eventually, under a corrosive environment, the grain boundary corrodes quickly and resulting in grain boundary cracking of the alloy.

T7 tempering (solution heat treatment then overaging) is recommended to lower the susceptibility of high strength Al-Cu-Mg (2XX series) or Al-Zn-Mg-Cu (7XXX series) alloys to SCC [1]. In the AA7075 (Al-Zn-Mg-Cu) alloy, for example, the primary strengthening phase is η (MgZn_2_), which has the lowest potential compared to the α matrix and PFZ [18]. The T7 temper coarsens the precipitation, resulting in a discontinuous structure along the grain boundary, thereby decreasing the alloy’s susceptibility to SCC [19]. However, for a high strength Al-Cu-Mg alloy, PFZ has the lowest potential than the CuAl_2_ and α matrix. The inhibition of the formation of PFZ is the primary way to improve the resistance of the Al-Cu-Mg alloy to SCC [20].

Although the effect of aging on the SCC behavior of high strength aluminum alloys had been investigated for decades [13,14,15,16,17,18,19,20,21], and these works mainly focused on the effect of single artificial aging on the SCC, such as T6 (peak aging) or T7 (over aging) treatment. However, the lack of research on multiple heat treatments (combined natural aging with artificial aging) is the primary purpose of this work. Hence, the influence of natural aging on the SCC behavior, microstructure, and mechanical properties of A201-T7 alloy were investigated in this work to find the feasibility of multiple heat treatments. The results can provide a reference for the development of high-performance alloys with lower SCC suspicious while the mechanical properties could be maintained.

## 2. Materials and Methods

### 2.1. Melting and Casting

The specimens for testing were prepared as follows. High purity aluminum ingots (99.9 wt.%) were first melted in an electric resistance furnace using a graphite crucible. Suitable amounts of pure Cu, Mg, Ag, Al-75Mn, and Al-60Ti master alloys were then added. Pure argon was used for degassing the melt for 40 min. After being held for 10 min at 700 °C, it was poured into a 125 mm × 100 mm × 25 mm steel mold preheated to 300 °C. Table 1 shows the experimental alloy’s chemical composition as determined by inductively coupled plasma optical emission spectrometry.

### 2.2. Heat Treatment of As-Cast Alloys

Table 2 shows the heat treatment cycles used to produce the as-cast alloy. First, they were solution treated at 510 °C for 2 h and then 530 °C for 20 h. Subsequently, water quenched (WQ) and immediately subjected to natural aging for different lengths of time (unaged, 24 h, 96 h). Finally, artificial aging was performed with T7 tempering at 190 °C for 5 h.

### 2.3. Slow Strain Rate Test

Slow strain rate testing (SSRT) was carried out in a 3.5% NaCl solution at pH = 7 using a tensile testing rate of 1.25 × 10^−7^ s^−1^. The test specimens prepared according to ASTM B557M [22] had dimensions of 4mm in diameter and 20 mm gauge length. The occurrence of suspected stress corrosion cracking was determined from the ductility loss rate [(E_scc_ − E_air_)/E_air_)] and ultimate tensile strength loss rate [(UTS_scc_ − UTS_air_)/UTS_air_)]. This is the ratio of elongation and tensile strength in 3.5% NaCl compared to that in air.

### 2.4. Materials Characterization

A Sigmascope SMP10 electrical conductivity meter was used to measure the alloys’ electrical conductivity under different heat treatment conditions. The unit of electrical conductivity were percentages of the international annealed copper standard (%IACS). Sample disks 3 mm in diameter and 1mm thick were prepared for the differential scanning calorimetry (DSC) experiments. A SEIKO-DSC6200 differential scanning calorimeter (Chiba, Japan) at a heating rate of 10 °C/min was used to capture the DSC traces. The T7 state specimens were prepared for transmission electron microscopy (FEI-TEM, Tecnai, G2-F20, Hillsboro, OR, USA) by twin-jet electro-polishing in a solution of 30% HNO_3_ methanol at −30 °C and 12 V. SSRT fractography was observed by scanning electron microscopy (FEI-SEM, JEOL, JSM-7800F Prime, Akishima, Japan). The Rockwell hardness B scale was used to measure the hardness of the experimental alloys.

### 2.5. Polarization Testing

Anodic potentiodynamic polarization experiments were performed in a 3.5% NaCl solution to determine the breakdown potentials of A201-T7 alloys prepared with various natural aging times. Silver-Silver chloride was used as the reference electrode and platinum as the auxiliary electrode. Each 15 mm diameter sample (working electrode) was exposed to the solution for 48 h before the start of the measurement and then potentiodynamically polarized from −630 mV below the open circuit potential (OCP) to a potential above the breakdown potential at 0.5 mV·s^−1^. The corrosion rates and polarization resistance were determined by the polarization resistance method and Stern-Geary equation. Besides, the polarization test was repeated three times in order to check the reproducibility.

## 3. Results and Discussion

### 3.1. Electrical Conductivity

As shown in Table 3, the alloys’ electrical conductivity decreased with an increase in the natural aging time. It was because the clustering of solute atoms led to a lattice distortion of the α matrix [23]. The longer the natural aging time was, the worse the lattice distortion became. As a result, the electrical conductivity of the alloys became lower. The changes in conductivity [(CAN−C_0_)/C_0_ × 100%] of the NA1d and NA4d alloys of 5.5% and 6.2%, indicated the flattening of clustering with an extension of the aging time from 24 h to 96 h.

### 3.2. DSC Analysis

**Figure 1:**
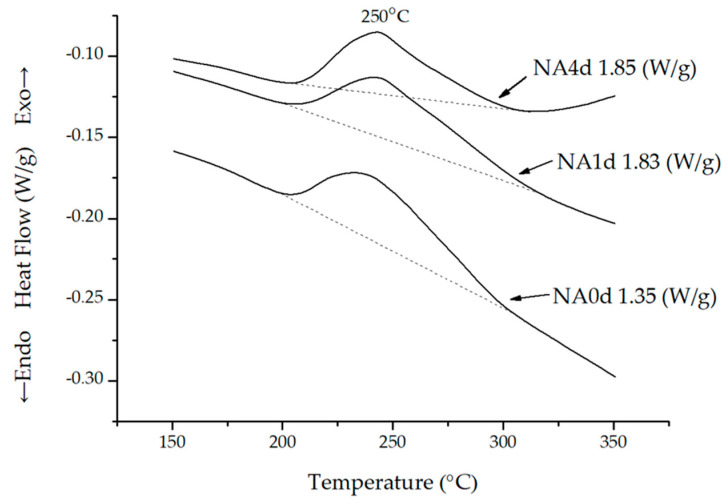
Differential scanning calorimetry (DSC) profile of different naturally aged A201 alloys.

An enlarged view of the DSC traces for the experimental alloys in the range of 150 °C to 350 °C is presented in Figure 1. The exothermic peak at 250 °C represents the precipitation of θ’ and Ω strengthening phase [24]. The enthalpy changes of the NA0d, NA1d, and NA4d alloys were 1.35, 1.83, and 1.85 W/g, respectively, showing that natural aging could significantly increase the exothermic heating. However, there was no obvious increase in exothermic heat while the increased period of natural aging time from 24 h to 96 h.

### 3.3. Mechanical Properties

The mechanical properties of the alloys produced under different heat treatment conditions are shown in Table 4. The hardness of the alloy after solution treatment then water quenching was 33.5HRB, which increased gradually following natural aging. The percentage changes [(H_NA_−H_0_)/H_0_ × 100%] of the alloys, NA1d and NA4d, were 40% and 54%, indicating that there was no obvious promotion of the clustering of solute atoms with an increase in the natural aging time from 24 h to 96 h, which was consistent with the electrical conductivity and DSC analysis results.

After T7 tempering, with the precipitation of the strengthening phases θ’ and Ω, there was an increase in the hardness of all the alloys, NA0d, NA1d, and NA4d, to 71HRB. Evidenced that the clustering of the solute atoms remained at the same level regardless of whether natural aging was adopted or not, and that lattice distortion would be eliminated after T7 tempering. The percentage changes [(H_T7_−H_NA_)/H_NA_ × 100%] of the alloys, NA0d, NA1d, and NA4d, were 112%, 53%, and 30%. Obviously, the hardness did not increase as much with the extension of the natural aging time from 24 h to 96 h. In addition, there was no difference in the yielding stress (YS), ultimate tensile stress (UTS), or elongation (EL) whether natural aging was adopted or not. The YS, UTS, and EL of the three alloys were approximately 320 MPa, 397 MPa, and 3.5%, respectively, after T7 tempering.

### 3.4. Polarization Test

**Figure 2:**
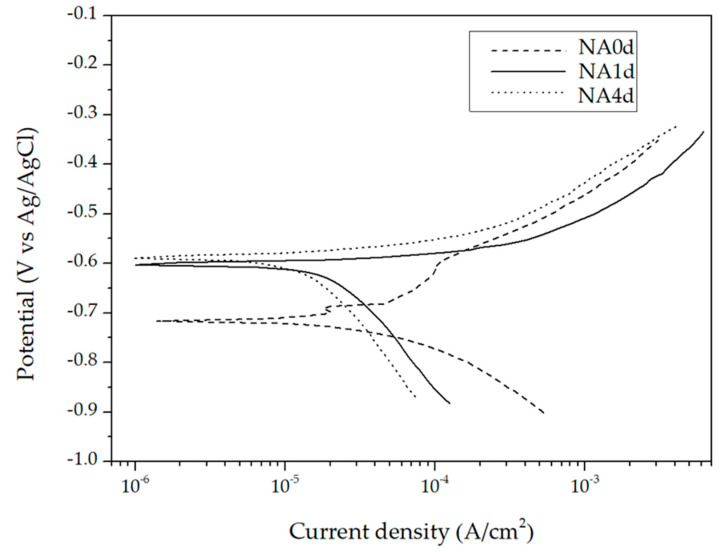
Polarization curves of different naturally aged A201-T7 alloys in a 3.5% NaCl solution.

The polarization curves of A201-T7 alloys after different natural aging (unaged, 24 h, 96 h) are shown in Figure 2. No passivation areas could be found after the samples were immersed in a 3.5% NaCl solution for 48 h before the test. The electrochemical analysis results were shown in Table 5. The corrosion potential, rates, and polarization resistance were represented as E**_corr_** (V), I_corr_ (A/cm^2^), and R_p_ (Ω/cm^2^), separately. The unaged alloy exhibited a lower corrosion potential and lower corrosion rates than the aged alloys. The corrosion potential of the NA0d alloy was −0.71 V, lower than for NA1d (−0.60 V), and NA4d (−0.59 V). The corrosion rates of the alloys, NA0d, NA1d, and NA4d, were 3.91 × 10^−5^, 5.94 × 10^−5^, and 6.77 × 10^−5^ (A/cm^2^), respectively, as determined by the polarization resistance method. Correspondingly, the polarization resistance of the naturally aged alloys was lower than that of the unaged alloy due to the clearly seen of active region in Figure 2. It is worth noting that the SCC resistance may be decreased with the long-term experiment. The polarization resistance of the unaged allot was 828 (Ω/cm^2^), while the 24 h and 96 h aged alloy were 633 and 604 (Ω/cm^2^). However, there was no obvious decrease in the polarization resistance of the A201-T7 alloys with an increase in the natural aging time from 24 h to 96 h.

### 3.5. Slow Strain Rate Testing

The results of slow strain rate testing of the A201-T7 alloys are presented in Table 6. Natural aging did not affect their elongation in air, approximately 3.5% for all three alloys. However, when it came to the saltwater environment, the elongation of the NA0d alloy decreased to 2.6%. In comparison, that of the NA1d and NA4d alloys remained about 3.5%, indicating that natural aging could significantly improve the alloy’s resistance to SCC. The losses of ductility of NA1d and NA4d were 5.4% and 5.7%, respectively, while the loss in the unaged alloy could be as much as to 27.8%. Moreover, the loss of strength also showed the same tendency. The loss of strength of the unaged alloy was 15%, while the 24 h and 96 h aged alloys showed losses of 2.5% and 3.0%, respectively.

The SSRT results indicated that natural aging before T7 tempering was essential, for it had the great benefit of increased resistance of the A201-T7 alloy to SCC. It also showed that aging for 24 h was sufficient. Extending the aging time further had no additional benefit. We would also like to remind the reader that the slow strain rate testing (SSRT) might not be suitable to determine the SCC behavior of alloys in the latest research due to the sub-critical cracking and to invalidate the SSRT results [25,26].

**Figure 3:**
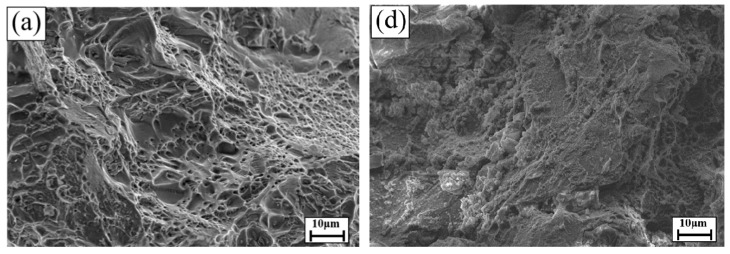

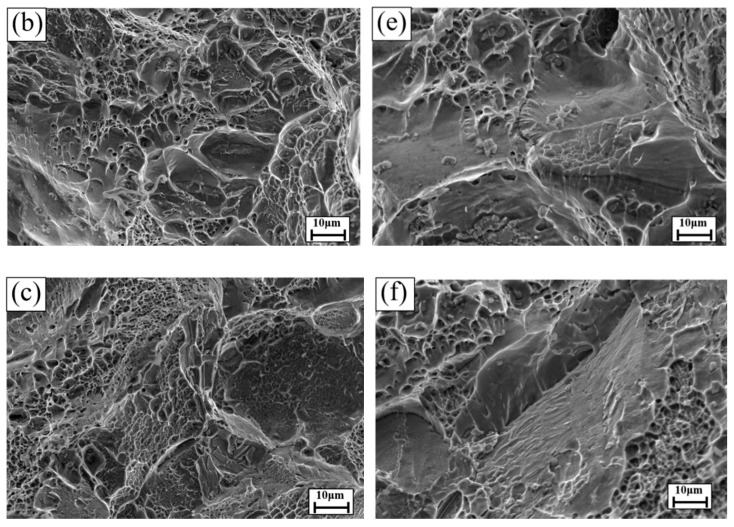
Fracture surface of A201-T7 alloys after slow strain rate testing: (**a**) NA0d sample in air; (**b**) NA1d sample in air; (**c**) NA4d sample in air; (**d**) NA0d sample in salt water; (**e**) NA1d sample in salt water; (**f**) NA4d sample in salt water.

An examination of Figure 3 shows the fracture surface of the A201-T7 alloys after slow strain rate testing. During SSRT in air, the fracture surfaces of the alloys, NA0d, NA1d, and NA4d, were similar with many dimples of different shapes and sizes observed, implying that the fracture mechanism was ductile fracturing. As a result, natural aging did not affect the elongation in the air. However, the fracture surfaces of the alloys were quite different when SSRT was conducted in the 3.5% NaCl solution. For the unaged alloy, nearly no dimples were observed, and the fracture mechanism was brittle fracturing. For the 24 h and 96 h aged alloys, cleavages and dimples were observed in the sample, and the fracture mechanism was a combination of ductile and brittle fracturing. The results were consistent with the mechanical properties presented in Table 4.

### 3.6. Transmission Electron Microscopy (TEM) Characterization

**Figure 4:**
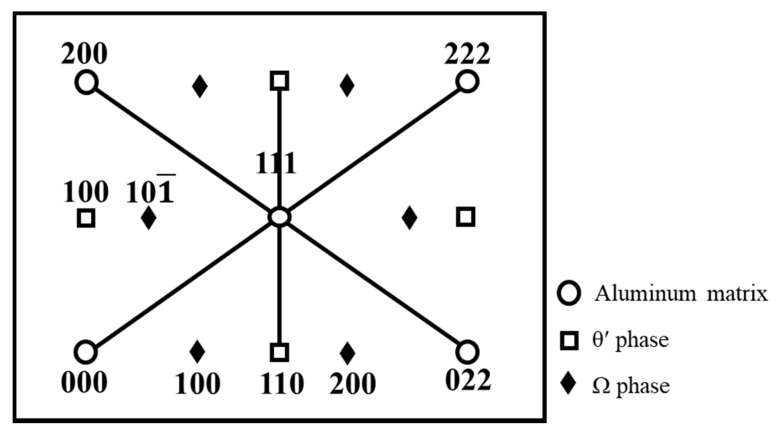
Schematic diagram of the diffraction pattern of θ’ and Ω strengthening phases along [011]_α_ zone in A201-T7 alloy.

**Figure 5:**
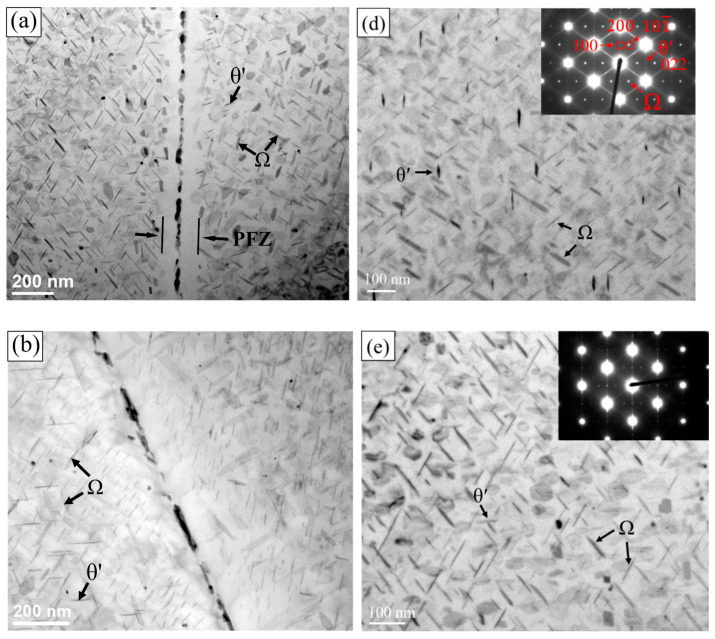

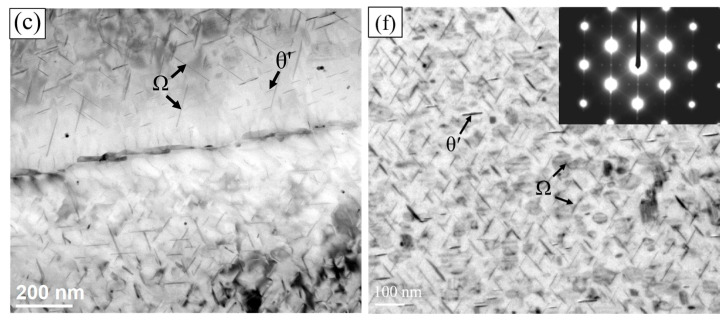
TEM bright field images and the selected area electron diffraction pattern (SAED) of: (**a**,**d**) Alloy NA0d; (**b**,**e**) Alloy NA1d; (**c**,**f**) Alloy NA4d after T7 tempering.

The Schematic diagram of the calculated diffraction pattern of Ω and θ’ strengthening phases along [011]_α_ zone axis were shown in Figure 4 [27]. Strengthening phases Ω and θ’ have a similar composition to CuAl_2_, but with a different crystal structure. As shown in Figure 5d, the selected area electron diffraction patterns (SAED) along [011]_α_ zone axis evidenced Ω and θ’ precipitation strengthening phases exist in A201 alloys after T7 tempered. The bright streaks and (10$\bar{1}$) diffraction spot of Ω phase and (100) diffraction spot of θ’ phase were found at (200)_α_ position. The TEM bright field images of the grain boundaries of A201-T7 alloys shown in Figure 5 reveal the presence of precipitation at the grain boundaries. The needle-like strengthening phases Ω and θ’ inside the grains can also be seen. The primary precipitation phase of A201-T7 alloys was Ω phases, and minor θ’ phases with different orientation can also be observed. It seems that natural aging did not affect the size of precipitation phases, Ω and θ’, after T7 heat treatment, just as shown in Figure 5d–f. The maximum length of Ω and θ’ phase of three different natural aged alloys (unaged, 24 h, 96 h) were around 100 nm and 70 nm, separately. The NA0d alloy had significant prefer-precipitation during T7 tempering [28]. The oversaturation of solute atoms near the grain boundaries would diffuse to the grain boundaries, forming coarse and discontinuous precipitation phases along with a 200 nm wide precipitation free zone (PFZ) as shown in Figure 5a. However, for NA1d and NA4d, no obvious PFZ could be found at the grain boundaries, as shown in Figure 5b,c. This might be due to the clustering of solute atoms (Cu, Mg), which occurred during natural aging, which would directly transfer to the precipitation phases (Ω and θ’) during T7 tempering, thus inhibiting the formation of PFZ.

It is worth noting that the PFZ has the lowest potential compared to CuAl_2_ phases (θ’ and Ω) and α matrix in the 2XX series and 2XXX series Al-Cu-Mg alloys [29]. To lower the galvanic corrosion effect, the inhibition of the formation of PFZ can help prevent SCC [30]. The discussion above is supported by the polarization test and slow strain rate test results, indicating that naturally aged alloys have better SCC resistance than unaged alloys.

## 4. Conclusions

The effects of natural aging on stress corrosion cracking in A201-T7 alloy were investigated in this study and the following conclusions can be drawn:

(1) For the as-quenched alloy, the conductivity decrease and the hardness increase during natural aging. However, the conductivity and mechanical properties (hardness, strength, and elongation) were unaffected by natural aging after T7 tempering.
(2) Natural aging improved the resistance of A201-T7 alloys to SCC. 24 h aging was sufficient. Extending the aging time provided no additional benefit.
(3) In the unaged alloys, PFZ existed and brittle fractures could be found on the SCC fracture surface; for the aged alloys, no PFZ existed, but a combination of fracture types with cleavages and dimples could be observed on the fracture surface.

## Figures and Tables

**Table 1:** Chemical composition of the experimental alloy (wt.%)**.**

Alloy	Cu	Mg	Ag	Ti	Fe	Si	Al
A201	4.5 (0.1) *	0.3 (0.05)	0.7 (0.05)	0.3 (0.05)	0.05 (0.01)	0.03 (0.01)	Balance

* Standard deviations are listed in parentheses.

**Table 2:** Heat treatment cycles for preparation of as-cast alloys.

Alloy Notation	Solution Treatment	Natural Aging	Artificial Aging
NA0d	510 °C/2 h + 530 °C/20 h + WQ	none	190 °C/5 h
NA1d	510 °C/2 h + 530 °C/20 h + WQ	24 h	190 °C/5 h
NA4d	510 °C/2 h + 530 °C/20 h + WQ	96 h	190 °C/5 h

**Table 3:** Electrical conductivity of alloys under different heat treatment conditions.

Alloy Notation	IACS (%)			Percentage Change (%)	
	As-Quenched (C_0_)	Natural Aging (C_NA_)	T7 (C_T7_)	$\frac{C_{NA}-C_{0}}{C_{0}}\;\times \;100\%$	$\frac{C_{T7}-C_{NA}}{C_{NA}}\;\times \;100\%$
NA0d	30.54 (0.12) *	30.54 (0.12)	32.54 (0.24)	-	6.5
NA1d	30.54 (0.12)	28.85 (0.11)	32.52 (0.11)	−5.5	12.7
NA4d	30.54 (0.12)	28.65 (0.09)	32.45 (0.09)	−6.2	13.4

* Standard deviations are listed in parentheses.

**Table 4:** Mechanical properties of alloys under different heat treatment conditions.

Alloy Notation	Hardness		Tensile Test				Percentage Change (%)	
	As-Quenched	Natural Aging	T7				$\frac{H_{NA}-H_{0}}{H_{0}}\;\times \;100\%$	$\frac{H_{T7}-H_{NA}}{H_{NA}}\;\times \;100\%$
	H_0_ (HRB)	H_NA_ (HRB)	H_T7_ (HRB)	YS (MPa)	UTS (MPa)	EL (%)		
NA0d	33.5 (2.2) *	33.5 (2.2)	71.1 (2.1)	320 (2.6)	398 (2.7)	3.5 (0.2)	-	112
NA1d	33.5 (2.2)	46.8 (1.5)	71.6 (1.6)	325 (2.0)	396 (2.8)	3.6 (0.1)	40	53
NA4d	33.5 (2.2)	54.5 (1.7)	70.6 (2.5)	318 (3.2)	397 (3.0)	3.5 (0.2)	54	30

* Standard deviations are listed in parentheses.

**Table 5:** Parameters of polarization test of different naturally aged A201-T7 alloys in a 3.5% NaCl solution.

Alloy Notation	E_corr_ (V)	I_corr_ (A/cm^2^)	R_p_ (Ω/cm^2^)
NA0d	−0.71 (0.05) *	3.91 × 10^−5^ (1.2 × 10^−5^)	828 (61)
NA1d	−0.60 (0.04)	5.94 × 10^−5^ (3.3 × 10^−6^)	633 (35)
NA4d	−0.59 (0.05)	6.77 × 10^−5^ (2.8 × 10^−6^)	604 (28)

* Standard deviations are listed in parentheses.

**Table 6:** Slow strain rate testing results of A201-T7 alloys.

Alloy Notation	EL in Air E_air_ (%)	EL in Salt Water E_scc_ (%)	UTS in Air UTS_air_ (MPa)	UTS in Salt Water UTS_scc_ (MPa)	$\frac{E_{scc}-E_{air}}{E_{air}}\times \;100\%$	$\frac{UTS_{scc}-UTS_{air}}{UTS_{air}}\;\times \;100\%$
NA0d	3.6 (0.1) *	2.6 (0.3)	399 (2.9)	339 (2.1)	−27.8	−15.0
NA1d	3.7 (0.2)	3.5 (0.2)	395 (2.6)	385 (2.8)	−5.4	−2.5
NA4d	3.5 (0.1)	3.3 (0.1)	397 (2.8)	385 (2.3)	−5.7	−3.0

* Standard deviations are listed in parentheses.
